# Substance P, A Promising Therapeutic Target in Musculoskeletal Disorders

**DOI:** 10.3390/ijms23052583

**Published:** 2022-02-26

**Authors:** Kyung Rae Ko, Hyunil Lee, Soo-Hong Han, Wooyeol Ahn, Do Kyung Kim, Il-Su Kim, Bo Sung Jung, Soonchul Lee

**Affiliations:** 1 Department of Orthopedic Surgery, Samsung Medical Center, Sungkyunkwan University School of Medicine, 81 Irwon-ro, Gangnam-gu, Seoul 06351, Korea; krmd.ko@gmail.com (K.R.K.); ilsu1.kim@samsung.com (I.-S.K.); 2 Department of Orthopedic Surgery, Ilsan Paik Hospital, Inje University, 170 Juhwa-ro, Ilsanseo-gu, Goyang-si 10380, Gyeonggi-do, Korea; hyunil.lee7@gmail.com; 3 Department of Orthopedic Surgery, CHA Bundang Medical Center, CHA University School of Medicine, 335 Pangyo-ro, Bundang-gu, Seongnam-si 13488, Gyeonggi-do, Korea; hsoohong@cha.ac.kr (S.-H.H.); a206032@chamc.co.kr (W.A.); fkzhfjqm@naver.com (D.K.K.)

**Keywords:** substance P, NK-1 receptor, musculoskeletal disorders, tendinopathy, rheumatoid arthritis, osteoarthritis, osteoporosis

## Abstract

A large number of studies have focused on the role of substance P (SP) and the neurokinin-1 receptor (NK1R) in the pathogenesis of a variety of medical conditions. This review provides an overview of the role of the SP-NK1R pathway in the pathogenesis of musculoskeletal disorders and the evidence for its role as a therapeutic target for these disorders, which are major public health problems in most countries. To summarize, the brief involvement of SP may affect tendon healing in an acute injury setting. SP combined with an adequate conjugate can be a regenerative therapeutic option in osteoarthritis. The NK1R antagonist is a promising agent for tendinopathy, rheumatoid arthritis, and osteoarthritis. Research on the SP-NK1R pathway will be helpful for developing novel drugs for osteoporosis.

## 1. Introduction

Musculoskeletal disorders (MSDs) are a heterogeneous group of conditions affecting the muscles, tendons, and bones in the spine and limbs. They represent a major public health problem, and their burden is increasing in most countries [1,2]. In addition to their own problems, they are accompanied by disorders in other medical fields such as sleep disorders and psychological problems [3,4].

Classic treatments for MSDs, based on antalgic drugs and passive physiotherapy, are often insufficient. More advanced treatments also exist, which can lead to the lasting regeneration of the musculoskeletal structures [5]. Clinical applications of extracorporeal shockwave therapy have been described in the treatment of MSDs, including the upper and lower extremity tendinopathies. However, no standardized protocol exists for the treatment [6]. Platelet-rich plasma (PRP) is also increasingly accepted for various MSDs due to its theoretical potential to repair tissues, but its effect is inconsistent [7]. Owing to the limitations of current therapies, new strategies such as stem cell or gene therapy are now being studied and developed for the treatment of MSDs. However, there is a lack of evidence to support their practical use in humans [8,9]. In other words, novel treatments are still needed for MSDs.

With this background, the purpose of this review is to update the findings that support the role of the substance P (SP)-related pathway in human MSDs. Recent publications on the corresponding issue in tendinopathy, rheumatoid arthritis, osteoarthritis, and osteoporosis, which are common MSDs in most countries [10], are reviewed.

## 2. Substance P

SP is an undecapeptide (i.e., 11-amino acid long peptide) which is encoded by the pre-protachykinin-A (TAC1) gene. SP belongs to the tachykinin neuropeptide family [11].

SP is synthesized and secreted by nerve cells. Monocytes, macrophages, dendritic cells, eosinophils, lymphocytes, and mast cells also synthesize SP [12]. Many studies have reported a widespread distribution of SP in the mammalian central and peripheral nervous systems [13,14,15]. Many studies have also reported a widespread distribution of its receptor in the mammalian central nervous system as well as the peripheral tissues [16,17,18]. In addition, SP exists in bodily fluids including the blood and cerebrospinal fluid. It mediates the crosstalk between the nervous and immune systems and regulates cell functions by diverse mechanisms (autocrine, paracrine, endocrine, and neuroendocrine) [19].

The enteric nervous system is composed of about 10^8^ neurons, which include a plethora of peptidergic neurotransmitters. The gastrointestinal (GI) mucosa is abundant with peptidergic innervation and immunocyte contents, thus providing the ideal environment for neuro–immune interactions. The tachykinins are expressed on the smooth muscle cells throughout the GI tract [20]. Among the members of the tachykinin neuropeptide family, SP regulates the contractility of the smooth muscle cells, vascular permeability, and the immune function as a gut hormone in particular [21,22].

SP is widespread throughout the human body. Further, it plays an important role in many physiologic and pathophysiologic functions such as inflammation, angiogenesis, and pain [23]. SP contributes to leukocyte recruitment in inflammatory processes. This recruitment involves the upregulation of adhesion molecule expression in endothelial cells and augments chemokine production or chemotaxis in leukocytes. Leukocytes can trigger endogenous antinociception through the release of opioid peptides and the activation of opioid receptors on peripheral sensory neurons [24]. Due to these various roles, the SP-related pathway has been proposed as a therapeutic target and its beneficial effects have been reported in a variety of medical fields including emesis, seizures, neurodegeneration, viral and bacterial infection, pruritus, wound healing, cancer, etc. [23,25,26,27].

## 3. Neurokinin-1 Receptor

Tachykinins are operated via three receptors, which are known as neurokinin (NK)-1, NK-2, and NK-3 receptors [16]. Among these, the NK-1 receptor (NK1R) has the highest affinity for SP [19]. After the ligand binding of SP to the NK1R, they are internalized into endosomes, and the clathrin-dependent internalization of the receptor is induced. After that, SP is degraded and the NK1R is recycled to the cell surface [28,29,30]. The SP–NK1R binding generates second messengers and triggers numerous mechanisms of the cell function [19,31,32]. Activation of the NK1R increases the intracellular Ca^2+^ and cyclic adenosine monophosphate (cAMP) concentrations, resulting in the Ca^2+^ increasing gene transcription and the cAMP-response element-binding protein [33].

There are more than 300 NK1R antagonists. They consist of two groups: (1) peptide NK1R antagonists, also known as SP antagonists, SP analog antagonists, and SP receptor antagonists, and (2) nonpeptide NK1R antagonists (L-733,060, L-741,671, L-742,694 (benzylether piperidines); RP-67,580, RP-73,467, RPR-100,893 (perhydroisoindolones); WIN-51,708 (steroid); L-732,138 (tryptophan-based); CP-99,994, CP-122,721, GR-203,040, GR-205,171 (benzylamino piperidines); CP-96,345, L-709,210 (benzylamino and benzylether quinuclidine)) [34].

The probability of experiencing side effects from the NK1R antagonists may be low, since peptide antagonists commonly act on altered systems with an increased peptide release [35]. In fact, the absence of serious side effects has been reported in many clinical trials of the NK1R antagonists administered to humans [36], even if they were administered at high doses (300 mg/day) [37]. These minor concerns about side effects may motivate us to conduct future studies on the NK1R antagonist.

The SP-NK1R pathway is upregulated in many human pathologies, suggesting that the use of the NK1R antagonists is a feasible strategy for the treatment of various diseases [23]. In fact, aprepitant and fosaprepitant are NK1R antagonists, which are used for acute and delayed chemotherapy-induced nausea and vomiting. In addition, NK1R antagonists have been used to block cardiac fibrosis, postoperative intra-peritoneal adhesions, fibrogenic factors in colitis-induced fibrosis, and TGF-β1 production by lung epithelial cells [38,39,40,41]. Similarly, recent studies have reported the potential for the use of NK1R antagonists for MSDs in humans (Table 1).

## 4. Tendinopathy

**Figure 1:**
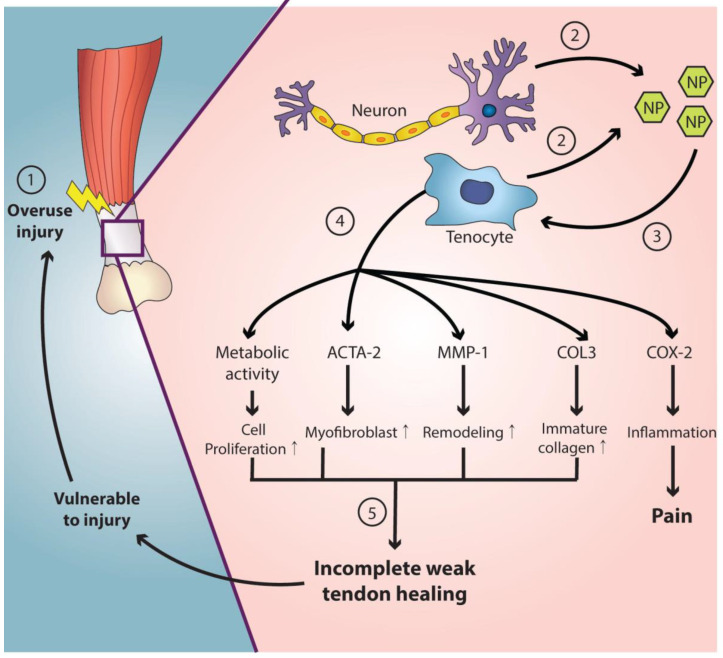
The etiology of tendinopathy. (1) The overuse (repetitive or excessive loading) and decreased local vascular perfusion are the acquired local factors [5]. (2, 3) Tenocytes produce signal substances including substance P, glutamate, catecholamines, and acetylcholine [47,48]. (4) Hypercellularity, fibrogenic response, expression of matrix metalloproteinases, production of abnormal amounts of collagen type III, and the inflammatory process contribute to the pathogenesis of tendinopathy. (5) If tendon healing is incomplete, the tendon becomes vulnerable to further injury. NP neuropeptide; ACTA-2 actin alpha 2; MMP-1 matrix metalloproteinase-1; COL3 collagen type III; COX-2 cyclooxygenase-2.

Tendinopathy is characterized by pain around the tendon and impaired functions sometimes associated with swelling of the tendon [5]. It affects millions of Americans and costs billions of health care dollars every year [45]. The rotator cuff, Achilles tendon, tibialis posterior tendon, and patellar tendon are its common sites [46]. The etiology of tendinopathy is understood as a multi-factorial process (Figure 1) [5]. The etiological factors are classified into: (1) innate general (older age, male sex, etc.), (2) acquired general (nutrition, body composition, underlying diseases, etc.), and acquired local factors (decrease in local vascular perfusion, repetitive or excessive loading, etc.) [5]. Hypercellularity, fibrogenic response, expression of matrix metalloproteinases, production of abnormal amounts of collagen type III, and the inflammatory process also contribute to the pathogenesis of tendinopathy [5].

With respect to treatment, rather than complete rest, a slowly progressive tendon-loading program is recommended [49]. Activity modification to alleviate the repetitive or excessive loading is also important [50]. The current medical therapies are mostly palliative without long-term benefits and carry risks of side effects. The use of nonsteroidal anti-inflammatory drugs (NSAIDs) can cause serious side effects, including GI complications and cardiovascular events [51,52]. Repeated injections of steroids increase the risk of tendon rupture [53,54]. PRP is increasingly favored to treat tendinopathy, but its efficacy is inconsistent [7]. Surgery is occasionally recommended after a prolonged period of conservative management. However, the ideal indication is debated [55]. Novel treatment options including stem cell or gene therapy are now being investigated and developed to treat tendinopathy. However, there is a lack of evidence for practical use in humans [5].

SP and NK1R increase in the tendons and muscles with overuse [56,57,58,59], and it has been suggested that the SP-NK1R pathway induces collagen production in overuse tendinopathies [56]. Thus, the SP-NK1R pathway has been proposed as a therapeutic target for tendinopathy in several recent publications [56,60,61]. Andersson et al. in particular reported that exogenous SP injections in the paratenon accelerated the development of tendinosis-like changes (hypercellularity and angiogenesis) and resulted in a paratendinous inflammation in the Achilles tendon of a rabbit [56]. Han et al. compared human healthy tenocytes and those with tendinopathy (in patients with lateral epicondylitis). SP was exogenously injected into the healthy tenocytes to investigate its effects. As a result, similar changes in tendinopathy (cell shape, and gene and protein levels) were observed after the SP injection [60]. Clearly, it seems that SP has a distinct role in the pathogenesis of tendinopathy. Interestingly, SP may also have a role in the restoration of human tendons in acute injury settings [62,63]. Steyaert et el. similarly reported that the injection of SP into the paratendinous region after an Achilles tendon rupture in rats resulted in improved tendon healing with the enhancement of biomechanical properties such as stress at a maximal load and work to a maximal load [64]. It is thought that SP may promote early tissue proliferation that is vital in the initial tendon repair. On the other hand, its long-term upregulation may have negative effects on tendon healing and may lead to the development of tendinopathy [60,65]. Consequently, it can be hypothesized that inhibiting NK1R by using NK1R antagonists may block the early progression of or improve the changes in tendinopathy. However, to date, there is a little clinical evidence for the practical applications of NK1R antagonists for tendinopathy. Barbe et al. investigated whether the SP-NK1R pathway was related to sensorimotor decline and fibrogenic responses by using rodent models of overuse injury. A specific NK1R antagonist (L-732,138) was intraperitoneally administered during the final two weeks of a 3-week high repetition high force task. As a result, it was found that the treatment reduced the fibrogenic responses in the tendon. It was also found that motor decline, mechanical hypersensitivity, and cold temperature aversion underwent improvements [42]. Nevertheless, it is still unclear and to be confirmed whether the NK1R antagonist can reduce or improve changes in tendinopathy occurring in various anatomical sites in humans. The NK1R antagonist, a promising agent for tendinopathy in humans, should be explored in future research.

## 5. Rheumatoid Arthritis

Rheumatoid arthritis (RA) is the most common inflammatory arthritis. It is a systemic autoimmune disease that causes joint destruction, physical disability, and excess morbidity and mortality [66]. The cytokine balance in the synovial tissue and serum is skewed toward the overexpression of pro-inflammatory markers including the tumor necrosis factor-α (TNF-α), interleukin (IL)-1, IL-6, IL-8, IL-12, IL-17, and the macrophage-colony stimulating factor (M-CSF). This imbalance causes further infiltration of diverse inflammatory and immune cells in the synovium, and finally the destruction of the joint [67].

With respect to treatment, medical therapies for RA have recently undergone improvements in which the development of drugs has been carried out in parallel with a deeper understanding of its pathogenesis [66]. The current medical therapies for RA can be classified into the following categories: NSAIDs, steroids, disease-modifying antirheumatic drugs (DMARDs), including conventional synthetic DMARDs, biologic DMARDs, and targeted synthetic DMARDs. NSAIDs are used most frequently to alleviate the pain. However, they appear to have no effects on the long-term course. Steroids are primarily used to relieve symptoms prior to the onset of action of DMARDs but minimizing their use has been emphasized due to the side effects. Conventional DMARDs, such as methotrexate, hydroxychloroquine, leflunomide, and sulfasalazine preserve the structure and function of the joints by slowing down the inflammatory process. Recently, biologic agents have aimed to inhibit the inflammatory process by depleting B cells or by blocking the inflammatory cytokine pathways, such as TNF-α or IL-6. Early recognition and the availability of and intervention with these new medications has greatly improved clinical outcomes and has led to the preservation of joint functions in a large proportion of patients with RA [66,68]. However, despite the advances in the knowledge of the etiology of RA and the development of novel biologic agents, a substantial number of patients remain resistant and do not respond to the current biologic therapies [69]. In addition to the medical therapies, many RA patients need orthopedic surgery such as joint replacement arthroplasty. However, many of these patients have other comorbidities and an increased risk of perioperative complications [70]. In other words, a huge effort is still needed to improve our view of the pathogenesis in RA which could result in the development of new drugs.

SP is a neuro-inflammatory mediator which is produced by sensory nerve fibers and local inflammatory cells [71]. It plays a distinct role in the skeletal degeneration and damage which are induced by chronic inflammation [72]. Abnormal expressions of the SP-NK1R pathway in various inflammatory diseases provide clues for its involvement in the inflammatory processes [23]. Further, the association of SP with various inflammatory processes suggests its role in the pathogenesis of inflammatory arthritis such as RA. Specifically, O’Connor et al. found increased SP levels in the synovial fluid and serum in RA patients. An upregulated NK1R mRNA level was also found in RA synovial cells [73]. In addition, SP-positive nerve fibers have been found in the synovium [74], and SP enhances the proliferation of the synovial cells [75]. SP also induces the expression of vascular cell adhesion molecule-1 on the synovial cells in human RA [76]. Consequently, it can be hypothesized that the suppression of the pro-inflammatory effects by using NK1R antagonists may be a therapeutic option in RA patients.

Recently, Liu et al. reported the potential for the use of NK1R antagonists in RA patients. Fibroblast-like synoviocytes (FLSs) were isolated from the synovial tissues of the knee joint from healthy and RA donors. FLSs were incubated in the presence of an NK1R antagonist (aprepitant). It was found that NK1R expression was significantly increased in FLSs in RA patients compared to healthy controls. It was also found that treatment with aprepitant decreased the release of inflammatory factors in FLSs in RA [43]. To summarize the results, blocking the NK1R may be a novel therapeutic option for autoimmune-related inflammatory diseases such as RA and other inflammatory arthritis. Nevertheless, it is still unclear and to be determined if this NK1R antagonist can mitigate disease activity in RA that shows various clinical manifestations on a case-by-case basis. The NK1R antagonist, a promising agent for RA patients, should be studied to verify its effectiveness. In addition to its potential as a therapeutic target, the serum SP level was reported as an indicator of disease activity and subclinical inflammation in patients with RA [77].

On the other hand, Hong et al. showed that treatment with SP (not NK1R antagonists) significantly improved local mean arthritis scores and reduced the degradation of joint cartilage, inflammatory signs, and the invasion of inflammatory cells using mouse models of collagen-induced arthritis. Furthermore, the treatment with SP significantly decreased the size of spleens enlarged by excessive inflammation with changes in cytokine pathways (increased IL-10 levels, and decreased TNF-α and IL-17 levels). They concluded that these effects of SP might be related to the suppression of inflammatory responses in RA and, moreover, a blockade of its progression [78]. It is interesting that both SP and NK1R antagonists showed similar anti-inflammatory effects. Clearly, SP has an anti-inflammatory function by increasing IL-10 and decreasing TNF-α [79]. Regarding these contradictory results, we believe that the SP-NK1R pathway has a role as an immune modulator rather than as an excessive expressor in the pathogenesis of inflammatory diseases. Further research to identify more detailed mechanisms related to the SP-NK1R pathway is needed.

## 6. Osteoarthritis

Osteoarthritis (OA), also known as degenerative arthritis, is one of the most common musculoskeletal disorders worldwide that results in significant health, social, and economic problems. Denudation of articular cartilage and sclerosis of the subchondral bone occur with the progression of OA [80]. Various person-level risk factors for OA including sociodemographic characteristics (female sex, African American race), genetic predispositions, obesity, diet-related factors, and high bone density have been reported. In addition, specific shapes of the bone or joint, thigh muscle weakness, malalignment of the joint, participation in certain occupational or sports activities, and joint injury have been reported as joint-level risk factors for OA [81].

Treatment for OA basically includes disease education and exercise programs. Similar to other chronic diseases, lifestyle modifications offer the most promising avenue for prevention of OA [82]. Established medical therapies with clinical evidence for OA are as follows: (1) topical NSAIDs, (2) COX-2 inhibitors, (3) intra-articular (IA) corticosteroids, and (4) IA hyaluronic acid [83]. The progressive and debilitating nature of OA can cause intractable pain and significant functional deficits, potentially leading to surgery such as joint replacement arthroplasty [84]. In addition, recent developments in treatment have focused on the feasibility of stem cell-based therapies in patients with OA [85,86].

Recently, the possibility of medical modifications of the disease process in OA has emerged through new anti-inflammatory agents [87,88]. More specifically, it has become clear that OA is not a simple matter of mechanical damage to the articular cartilage of the joint. Further risk factors and related chemical pathways of inflammation have been reported. The biomechanical imbalance or joint injury results in local tissue damage with inflammation which is disseminated by the innate immune system. The damage-associated molecular patterns lead to the activation of immune-modulated mechanisms, resulting in the production of catabolic elements that can impair the native structures of the joint such as cartilage [87].

Cartilage was traditionally considered as inert tissue. In contrast, recent studies have shown that it is regulated by sensory nerves, and sensory neuropeptides are related to physiological or pathological metabolism by affecting the proliferation, differentiation, and secretion of chondrocytes [89,90,91]. Specifically, increased SP levels were found in the synovial fluid obtained from OA patients who underwent total knee replacement surgery, suggesting its catabolic effects on cartilage [92]. Saito et al. demonstrated increased SP-immuno-reactive nerve fibers using immunohistochemistry in patients with knee OA, suggesting that free nerve endings with SP may regulate and play an important role in the inflammation and pain pathways in OA [93]. Lisowska et al. reported that serum SP levels were positively associated with chronic pain intensity in OA and RA patients. More specifically, the serum SP level was significantly higher in RA patients compared to OA [94]. More recently, Okamura et al. cultured synovium-derived mast cells obtained from patients with RA or OA who underwent joint replacement arthroplasty and compared the SP expression localized around the cell membrane. The results showed that the activation of the mast cells was greater in RA (41% vs. 7% in OA) [95].

Lam et al. reported the potential for the use of NK1R antagonists in OA patients. They used rat models of arthritis, induced by a single injection of 125 µL Freund’s complete adjuvant (containing 125 µg Mycobacterium tuberculosis) into the knee joint. They reported that the NK1R antagonist (RP-67,580 was injected into ipsilateral knee joint) led to reduced arthritic pain and swelling of the knee joint in rats [44]. Of course, the corresponding rat model could not fully reflect OA of the knee joint in humans. Nevertheless, blocking NK1R using the antagonist may be a promising therapeutic strategy for OA. To summarize, it seems that high SP activations can be treated as evidence supporting the role of inflammation in the pathogenesis of OA, similar to RA. However, the relative importance of the SP-related inflammatory process may be lower in OA compared to RA.

The results of a pre-clinical study on the therapeutic application of SP with the specific hydrogel implant for OA are interesting and noteworthy. Kim et al. used the rat models and SAP conjugates (SP with self-assembled peptide). The SAP combined with SP significantly improved cartilage regeneration by recruiting mesenchymal stem cells (MSCs). The corresponding treatment could prevent apoptosis by secreting anti-inflammatory cytokines, promoting chondrogenesis and differentiation, and reducing inflammation [96]. It is especially interesting that SP showed anti-inflammatory and regenerative properties in the specific setting. Regarding these contradictory effects of SP (inflammatory vs. anti-inflammatory and regenerative properties), we believe that they can be explained by the modification of the dose, the continuity of release, and the use of an adequate conjugate [96]. The role of the SP-NK1R pathway as a therapeutic target for OA and its specific setting are topics that need to be studied further.

## 7. Osteoporosis

Osteoporosis is a common disorder of the bone metabolism. It results in bone deterioration, diminished bone density, and a heightened risk of skeletal fractures [97,98] Osteoporosis and the accompanying fractures have a considerable impact in terms of morbidity and mortality on individuals, healthcare systems, and communities [99]. The goals of therapy are the prevention of bone loss and mineral density replacement, thus minimizing the risk for fractures [100]. Current medical treatments for osteoporosis include bisphosphonates (alendronate, risedronate, ibandronate, and zoledronic acid), peptide hormones (teriparatide and calcitonin), estrogen for postmenopausal women, selective estrogen receptor modulators (raloxifene), RANK ligand inhibitor (denosumab), etc. Supplemental calcium and vitamin D are also used [101,102]. Although the above-mentioned medications are available, their side effects have raised safety concerns and been related to critical diseases such as bisphosphonate-related osteonecrosis of the jaw [103]. In other words, new drugs for osteoporosis still need to be developed.

Bone remodeling normally relies on the differentiation and activation of two types of cells with opposite functions. First, osteoclasts differentiate from hematopoietic stem cells and perform bone resorption. Second, osteoblasts, the major cellular component of bone, arise from MSCs and enhance bone formation [104,105]. Many studies have reported the role of inflammatory processes in physiologic bone remodeling and bone-related diseases such as osteoporosis. Osteoclast-induced bone resorption is especially mediated by diverse factors, including TNF-α, IL-1, and IL-7 [106,107]. Inflammatory conditions can promote bone resorption via the activation of osteoclasts [108], and aging-induced inflammation has been regarded as the main factor of bone microarchitecture impairment [109]. Estrogen deficiency after menopause causes the creation of an inflammatory microenvironment in the bone marrow via the accumulation of reactive oxygen species and inhibits bone remodeling [110]. Further, it has been reported that an imbalance between regulatory T (Treg) and T helper 17 (Th17) cells plays a distinct role in the progression of osteoporosis. The ratio of Treg/Th17 is commonly low in osteoporosis [111,112]. In addition to the inflammatory processes, angiogenesis is the vital process in bone remodeling and the synthesis of a normal osteoblastic bone matrix [113].

SP has various functions in the bone metabolism [23,108,114], suggesting that SP could be a therapeutic target for osteoporosis. SP is known to have therapeutic effects against inflammatory diseases via the suppression of inflammatory responses by increasing the number of Treg cells and decreasing the TNF-α level [78,115]. SP can stimulate the recovery of bone marrow stem cells [116]. It also protects the vascular endothelium and promotes angiogenesis [117,118,119].

The NK1R is expressed by osteoblasts, osteoclasts, and their precursors [120]. By binding to the receptor, neuropeptides such as SP control the functions of osteoclasts and osteoblasts, and participate in bone repair, reconstruction, and growth [114]. The accelerated effect of SP on osteoclastic bone resorption has previously been reported. On the other hand, the effect of SP on bone formation is still not fully understood [114]. Nevertheless, there have been many animal studies on the severely decreased expression of SP in bone marrow in animal models of osteoporosis, indicating its role in bone formation and the pathogenesis of osteoporosis [121,122]. More specifically, Liu et al. reported that an ovariectomy reduced SP in the bone in their adult female rat models of osteoporosis [123]. Kingery et al. suggested that a widespread reduction in SP content in the bone might lead to osteoporosis after a sciatic neurectomy by investigating rat models [124]. Chen et al. used gelatin microspheres consisting of different concentrations of SP. Regardless of the concentration of SP, the gelatin microspheres promoted osteogenesis based on the results of a histological analysis in rabbit models of osteoporosis [125]. Further, SP blocked the ovariectomy-induced impairment of the bone microarchitecture and a reduction in the mineral density in rats, suggesting its therapeutic effects on osteoporosis [126]. All of the above-mentioned studies are limited in that they only used animal models. Nevertheless, it seems that the pathophysiology of bone-related diseases such as osteoporosis is associated with the SP-NK1R pathway. We should focus on the SP-NK1R pathway in future research on osteoporosis.

## 8. Conclusions

To summarize this review, the brief involvement of SP may be linked to tendon healing in acute injury settings. In addition, SP combined with an adequate conjugate can be a regenerative therapeutic option in OA and other diseases with cartilage damage. The NK1R antagonist could be a promising agent for tendinopathy, rheumatoid arthritis, osteoarthritis, and other inflammatory arthritis. Research on the SP-NK1R pathway will be helpful for exploring the mechanism of drugs and for developing novel drugs for bone-related diseases such as osteoporosis. Referencing the therapeutic effects of both SP and NK1R antagonists in RA, we believe that the SP-NK1R pathway may act as an immune modulator rather than an excessive expressor in the pathogenesis of inflammatory diseases.

This review has demonstrated the role of the SP-NK1R pathway in various musculoskeletal disorders, which should be further investigated in the future. Future clinical trials are required to fully determine the safety and efficacy of SP and NK1R antagonists.

## Figures and Tables

**Table 1:** Recent studies on the use of neurokinin-1 receptor antagonists in musculoskeletal disorders.

Authors/Year	Barbe et al., (2020) [42]	Liu et al., (2019) [43]	Lam et al., (2010) [44]
Disease	Tendinopathy.	RA.	OA (mono-arthritis of the knee joint).
Model	Rodent model of an overuse injury (performance of a high repetition high force task for 3 weeks).	FLSs in RA patients.	Rat model of arthritis (a single intra-articular injection of 125 µL Freund’s complete adjuvant containing 125 µg Mycobacterium tuberculosis).
Human/Animal	Animal.	Human.	Animal.
Neurokinin-1 receptor antagonist	L-732,138 was intraperitoneally administered at a dose of 5 mg/kg for 3 days/week in task weeks 2 and 3 (the final two weeks of a 3-week task).	Aprepitant (FLSs were incubated in the presence of aprepitant (5, 10 μM) for 24 h).	RP-67,580 was injected into ipsilateral knee joint in in a final volume of 100 µL.
Effects	Reduced fibrogenic responses in the tendon, muscle, and dermal tissues. Motor declines, mechanical hypersensitivity, and cold temperature aversion underwent improvements.	(1) Reduced TNF-α-induced expression of NADPH oxidase 4 and generation of reactive oxygen species, (2) inhibited TNF-α-induced expression and secretion of proinflammatory cytokines, (3) prevented TNF-α-induced expression of MMPs, (4) inhibited TNF-α-induced phosphorylation, and (5) attenuated TNF-α-induced nuclear translocation.	Reduced pain and swelling of the knee joint.
Other information	n/a	n/a	Did not attenuate hyperemia or histological changes (polymorphonuclear cell infiltration, synovial tissue proliferation, and cartilage erosion) which were shown after treatment with dexamethasone.

RA rheumatoid arthritis; OA osteoarthritis; FLSs fibroblast-like synoviocytes; TNF-α tumor necrosis factor-α; MMPs matrix metalloproteinases; n/a not applicable.

## Data Availability

Not applicable.
